# Exploring the Microstructural Effect of FeCo Alloy on Carbon Microsphere Deposition and Enhanced Electromagnetic Wave Absorption

**DOI:** 10.3390/nano14141194

**Published:** 2024-07-12

**Authors:** Xiaoshu Jia, Heng Zhang, Fang Liu, Qiaojun Yi, Chaolong Li, Xiao Wang, Mingxing Piao

**Affiliations:** 1 Key Laboratory of Multi-Scale Manufacturing Technology, Chongqing Institute of Green and Intelligent Technology, Chinese Academy of Sciences, Chongqing 400714, China; 202209021022@stu.cqu.edu.cn (X.J.); zhangheng@cigit.ac.cn (H.Z.); yiqiaojun@stu.cqut.edu.cn (Q.Y.); lichaolong@cigit.ac.cn (C.L.); wangxiao@cigit.ac.cn (X.W.); 2 College of Material Science and Engineering, Chongqing University, Chongqing 400030, China; xiaoliu@yeah.net

**Keywords:** FeCo@CM composites, laminar-stacked FeCo alloy, MPARCVD technique, electromagnetic wave absorption

## Abstract

The rational design of magnetic carbon composites, encompassing both their composition and microstructure, holds significant potential for achieving exceptional electromagnetic wave-absorbing materials (EAMs). In this study, FeCo@CM composites were efficiently fabricated through an advanced microwave plasma-assisted reduction chemical vapor deposition (MPARCVD) technique, offering high efficiency, low cost, and energy-saving benefits. By depositing graphitized carbon microspheres, the dielectric properties were significantly enhanced, resulting in improved electromagnetic wave absorption performances through optimized impedance matching and a synergistic effect with magnetic loss. A systematic investigation revealed that the laminar-stacked structure of FeCo exhibited superior properties compared to its spherical counterpart, supplying a higher number of exposed edges and enhanced catalytic activity, which facilitated the deposition of uniform and low-defect graphitized carbon microspheres. Consequently, the dielectric loss performance of the FeCo@CM composites was dramatically improved due to increased electrical conductivity and the formation of abundant heterogeneous interfaces. At a 40 wt% filling amount and a frequency of 7.84 GHz, the FeCo@CM composites achieved a minimum reflection loss value of −58.2 dB with an effective absorption bandwidth (*f_E_*) of 5.13 GHz. This study presents an effective strategy for developing high-performance EAMs.

## 1. Introduction

With the rapid advancement of radar detection technology and fifth-generation (5G) electromagnetic communication technology, significant progress has been achieved for humanity. Nevertheless, this has also led to significant electromagnetic pollution and interference issues [1,2,3,4], posing formidable challenges to electronic information security, military security, and even human life and health [5,6,7,8]. Consequently, the development of electromagnetic wave-absorbing materials (EAMs) that adhere to the criteria of “thin, light, wide, and strong” holds immense significance [9,10,11,12]. 

Magnetic materials with high magnetization and permeability, including carbonyl iron [13], ferrite [14], and metals and their alloys [11], have been extensively employed owing to their proficiency in achieving high electromagnetic loss with reduced thickness [15]. In comparison to traditional ferrites, FeCo alloys have exhibited higher Curie temperatures, lower coercivity, greater magnetocrystalline anisotropy, and a higher Snoek’s limit, positioning them as promising candidates for EAMs [16,17,18,19]. Jiang et al. [20] synthesized Co_x_Fe_10−x_ alloy nanochains with varying iron molar fractions, utilizing NaBH_4_ as a reducing agent. Among these, the Co_4_Fe_6_ alloy displayed the most promising microwave absorbing performance, attaining a minimum reflection loss (RL_min_) of −38.7 dB with an effective absorption bandwidth (defined as RL < −10 dB, *f*_E_) of 6.9 GHz. Cheng et al. [21] successfully synthesized diverse morphologies of FeCo alloys with varying atomic ratios through a hydrothermal reduction process. Among these, Fe_7_Co_3_ exhibited optimal microwave absorption properties, achieving the RL_min_ of −53.6 dB at an *f*_E_ of 6.8 GHz. Yang et al. [22] prepared magnetic FeCo alloy materials with an evolved micro-scale succulent plant-like morphology through a simplified hydrothermal reaction–hydrogen reduction synthesis strategy involving multiple equilibrium/competition mechanisms, which attained an RL_min_ of −53.81 dB at an *f*_E_ of 5.68 GHz. However, as a sole magnetic loss material, FeCo alloy lacks a dielectric loss mechanism, which could contribute to its suboptimal impedance-matching performance [23]. Moreover, FeCo encountered challenges in oxidation resistance and thermal stability in a natural environment [24]. Additionally, the dense magnetic FeCo alloy faced practical application constraints due to the increasing demand for lightweight electronic devices [19]. To tackle these limitations, the optimal approach involved introducing dielectric loss components into the FeCo magnetic system [25,26,27,28], which capitalized on the synergistic and complementary dielectric–magnetic coupling effect between the magnetic and dielectric components [29,30,31]. 

Carbon materials, as typical dielectric loss materials, exhibit significant advantages, including lightness, chemical stability, and environmental friendliness [32,33]. Furthermore, the dielectric properties are highly tunable, rendering magnetic carbon composites, which combine carbon materials with magnetic components, as ideal EAMs [34,35,36]. Currently, high-temperature carbonization of organic materials is the prevalent method for obtaining carbon sources. For example, Wang et al. [37] synthesized porous CoFe@C nanorods with a hierarchical nested structure, utilizing CoFe-MOF-74 as a precursor through high-temperature pyrolysis. The multi-layered nested structures with multi-scale pores facilitated the enhancement of multiple reflections and diffractions of electromagnetic waves. Moreover, the presence of numerous interconnected FeCo@C nanorods within the multi-layered nested structures generated stronger conductive loss. When the molar ratio of Co/Fe was 3:1, the RL_min_ achieved −61.8 dB with an *f*_E_ of 9.2 GHz. Similarly, Wang et al. [38] employed FeCo Prussian blue analogues as nucleation sites, polymerized dopamine on their surfaces, and converted the precursor into ideal core–shell FeCo@C nanoparticles via high-temperature pyrolysis. The gradient crystallinity between the graphitic carbon shell and the amorphous carbon nanocages provided a simulated “bilayer” microstructure that favors the penetration of electromagnetic waves. The encapsulation of core–shell FeCo@C nanoparticles within the carbon nanocages potentially stimulated multiple reflection behaviors of incident electromagnetic waves, thereby enhancing the attenuation capabilities. When the weight ratio of DA to FeCo PBAs reached 0.75, the RL_min_ attained −67.8 dB with an *f*_E_ of 5.3 GHz. Based on various published research findings, it is evident that the electromagnetic wave absorption performance of FeCo@C composites is notably enhanced compared to single magnetic FeCo material components. Nonetheless, the preparation methods of these FeCo@C composites involved intricate processes, high energy consumption, and low yield, resulting in elevated production costs and impeding large-scale production for practical applications. Therefore, adopting a simpler, more energy-efficient, and cost-effective synthesis route for FeCo@C composites is of significant importance. 

In this study, an advanced microwave plasma-assisted reduction chemical vapor deposition (MPARCVD) technique was utilized to efficiently fabricate FeCo@CM composites with high efficiency, low cost, and energy-saving benefits. The deposition of graphitized carbon microspheres significantly enhanced the dielectric properties, leading to an improved electromagnetic wave absorption performance via enhanced impedance matching and a synergistic effect with the magnetic loss. Furthermore, a systematic investigation was conducted to examine the effect of the FeCo microstructure, specifically comparing spherical versus laminar structure, on the regulation of the dielectric properties of the FeCo@CM composites. It was demonstrated that the laminar-stacked structure of FeCo exhibited a higher number of exposed edges and increased catalytic activity, offering more energy sites for the deposition of carbon species. As a result, a large number of uniform and low-defect graphitized carbon microspheres were successfully deposited on the surface of the laminar microstructure of the FeCo alloy. The dielectric loss performance of the FeCo@CM composites was significantly improved due to the increased electrical conductivity and the newly formed abundant heterogeneous interfaces. Consequently, when the filling amount of the FeCo@CM composites reached 40 wt%, at a frequency of 7.84 GHz, the RL_min_ value achieved was −58.2 dB, accompanied by an *f*_E_ of 5.13 GHz. This study provided an effective strategy and means for acquiring high-performance EMAs. 

## 2. Materials and Methods

### 2.1. Materials

Ferric chloride hexahydrate (FeCl_3_·6H_2_O), cobalt chloride hexahydrate (CoCl_2_·6H_2_O), hexamethylenetetramine (HMT), and absolute ethanol (C_2_H_5_OH) were all procured from Alfa Aesar (Haverhill, MA, USA), while the iron–cobalt alloy powder (FeCo) with an atomic ratio of 1:1, labeled *s*-FeCo, was sourced from Qinghe County Hengbei Metal Materials Co., Ltd. (Xingtai, China). Additionally, H_2_ (99.999%), Ar (99.999%), and CH_4_ (99.999%) gases were obtained from Chongqing Ruixin Gas Co., Ltd. (Chongqing, China). All these reagents were utilized without any further purification. Deionized water, prepared using an ultrapure water machine (ATSelem 1810A), from Shanghai Zhong Zhuang Pure Water Technology Co., Ltd. in Shanghai, China, served as the medium throughout the entire experimental process. 

### 2.2. Preparation of f-Fe(OH)_3_/Co(OH)_2_ Precursor

Utilizing HMT as the hydrolyzing agent, the precursor *f*-Fe(OH)_3_/Co(OH)_2_ was synthesized via a hydrothermal approach. Initially, FeCl_3_·6H_2_O (2 mmol), CoCl_2_·6H_2_O (2 mmol), and HMT (96 mmol) were dissolved in a mixed solvent consisting of 70 mL water and ethanol (9:1, *v*/*v*) and agitated at room temperature for 20 min. Subsequently, the reaction solution was transferred to a 100 mL hydrothermal reactor and heated to 150 °C for 2 h. Following the reaction, the obtained dark brown precipitate was gathered, rinsed alternately with anhydrous ethanol and deionized water four times, and ultimately dried in an oven at 70 °C for 12 h, resulting in the acquisition of the *f*-Fe(OH)_3_/Co(OH)_2_ precursor.

### 2.3. Preparation of f-FeCo Alloy 

The FeCo alloy was synthesized by reducing the precursor using a homemade microwave plasma apparatus and was designated as *f*-FeCo. The apparatus comprised a household microwave oven, a vacuum controller, a vacuum pump, and a quartz tube. Upon activation, the device generated purple microwave plasma with an operating frequency of 2450 MHz and an output power of 700 W. A total of 0.5 g of the *f*-Fe(OH)_3_/Co(OH)_2_ precursor was positioned in a quartz boat and subsequently transported to the center of the quartz tube. Following the evacuation of the reaction chamber to a vacuum state, Ar (100 sccm) and H_2_ (100 sccm) were introduced into the furnace. Subsequently, the microwave oven switch was activated to initiate the reduction reaction. The reaction duration was set for 20 min, ensuring the complete reduction of *f*-Fe(OH)_3_/Co(OH)_2_ to *f*-FeCo alloy. After the reaction, the H_2_ flow was terminated, and the sample was cooled to room temperature under an Ar atmosphere.

### 2.4. Preparation of f-FeCo@CM and s-FeCo@CM

A total of 0.2 g of *f*-FeCo or *s*-FeCo alloy particles was positioned in a quartz boat, which was then placed at the center of a quartz tube. Once the reaction apparatus was evacuated to a vacuum state, Ar (100 sccm), H_2_ (200 sccm), and CH_4_ (100 sccm) were introduced into the furnace. Subsequently, the microwave oven switch was activated to commence the reaction, which proceeded for 20 min. During this process, carbon material was deposited onto the surfaces of FeCo alloys. Following the reaction, the flows of H_2_ and CH_4_ were terminated, and the samples were cooled to room temperature under an Ar atmosphere, ultimately yielding FeCo@CM samples named *f*-FeCo@CM or *s*-FeCo@CM, respectively.

### 2.5. Characterization

The phase structures of the samples were characterized by X-ray powder diffraction (XRD) patterns, which were acquired using an X-ray diffractometer (X’Pert 3 powder, PANALYTICAL, Almelo, The Netherlands) with Cu Kα radiation (λ = 1.54 Å). The surface morphology was observed with a field emission scanning electron microscope (FESEM, JSM-7800F, JEOL, Akishima City, Tokyo, Japan), while the elemental composition was determined via energy dispersive spectroscopy (EDS). Furthermore, Raman spectra were recorded using a laser Raman spectrometer (Raman, InVia Reflex, Renishaw, London, United Kingdom) with 532 nm laser excitation to assess the defect concentration and graphitization degree of the samples. The thermal stability of the samples in an air atmosphere was evaluated using a thermogravimetric analyzer (TGA DSC1, Mettler-Toledo Group, Zurich, Switzerland) at a scanning rate of 10 °C min^−1^ over a temperature range from room temperature to 1100 °C. Surface composition analysis was performed using X-ray photoelectron spectroscopy (XPS, Thermo ESCALAB-250Xi, Thermo Fisher Scientific, Waltham, MA, USA). The magnetization hysteresis curves were measured with a vibrating sample magnetometer (SQUID, MPMS XL, Quantum Design, San Diego, CA, USA). For electromagnetic parameter measurement, the samples were mixed with paraffin at a filling factor of 40 wt% and compressed into rings with an approximate thickness of 2 mm. The relative permittivity and permeability were then determined within the frequency range of 2–18 GHz using a vector network analyzer (N5234A, Agilent, Santa Rosa, CA, USA) via the coaxial method. The reflection loss value (RL) was calculated based on transmission line theory, expressed by the following equation:

(1) $$Z_{in}=Z_{0}\sqrt{\frac{\varepsilon_{r}}{\mu_{r}}}\tan h\left\{j\left(\frac{2\pi fd}{c}\right)\sqrt{\mu_{r}\varepsilon_{r}}\right\}$$

(2) $$RL=20\log\left|\frac{\left(Z_{in}-Z_{0}\right)}{\left(Z_{in}\;+\;Z_{0}\right)}\right|$$

Among them, *Z*_0_ and *Z_in_* are the free space impedance and the absorber input impedance respectively, *ε_r_* and *μ_r_* distribution are the complex dielectric constant and complex permeability, respectively, *f* is the electromagnetic wave frequency, *d* is the thickness of the absorber, and *c* is the speed of light.

## 3. Results and Discussion

**Figure 1:**
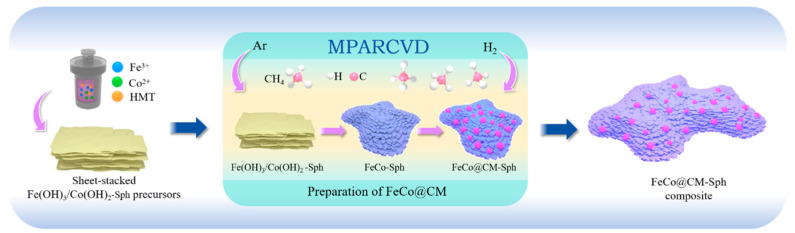
Schematic diagram of the synthesis process of *f*-FeCo@CM using MPARCVD technology.

The preparation process and the underlying reaction mechanism of *f*-FeCo@CM are delineated in Figure 1. Utilizing the MPARCVD technique, the magnetron in the microwave oven proficiently converted electrical energy into microwaves, efficiently inducing low-pressure H_2_ molecules to generate plasma, which comprised hydrogen ions (H^+^, H_2_^+^, H_3_^+^) and hydrogen atoms in both the ground (H) and excited (H*) states. As a highly potent reducing agent, hydrogen plasma possessed a greater activation energy than H_2_ molecules obtained through traditional thermal reduction [39], thereby significantly enhancing reduction efficiency. During the carbon growth phase, CH_4_ molecules decomposed under the influence of H_2_ plasma, where C-H bonds were disrupted by the etching action of the plasma. The liberated C atoms were subsequently adsorbed onto the FeCo substrate surface, seeking locations of minimum energy for deposition. According to the previous literature [40,41], the remarkable activity of Fe-Co bimetallic catalysts was demonstrated in the fabrication of carbon nanomaterials via the CVD method. Given that both Fe and Co lattices exhibited a certain solubility for C atoms, the combination of Fe and Co metals generated a synergistic effect, enhancing the affinity for C atoms and augmenting the diffusion coefficient of C atoms, thus promoting the efficient growth of carbon nanomaterials [42,43]. Therefore, the MPARCVD technique could efficiently achieve the reduction of the *f*-Fe(OH)_3_/Co(OH)_2_ precursor and the growth of carbon microspheres on *f*-FeCo/*s*-FeCo alloy particles with the assistance of H_2_ plasma, exhibiting high efficiency and energy-saving attributes.

**Figure 2:**
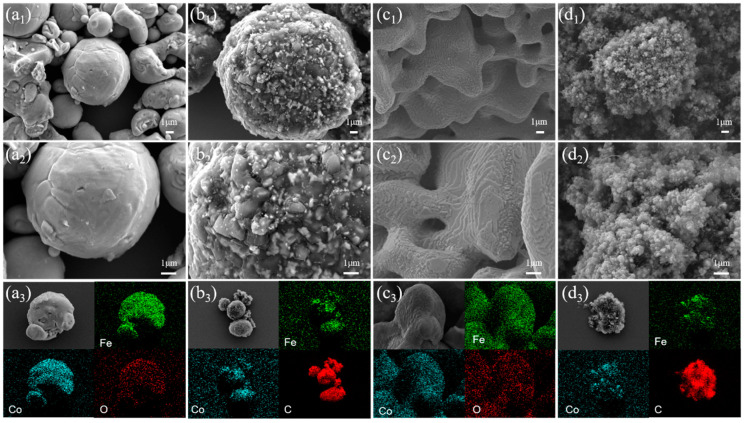
The FESEM images and corresponding elemental mapping of the samples: (**a_1_**–**a_3_**) *s*-FeCo; (**b_1_**–**b_3_**) *s*-FeCo@CM; (**c_1_**–**c_3_**) *f*-FeCo; (**d_1_**–**d_3_**) *f*-FeCo@CM.

The morphological analysis of the samples was performed using the FESEM. Initially, the low-magnification SEM images (Figure S1a,b) demonstrated that the *f*-Fe(OH)_3_/Co(OH)_2_ precursor exhibited irregular bulk particles, composed of numerous sheet structures that were challenging to discern visually. The Fe and Co elements within the precursor exhibited a uniform distribution, with an atomic ratio approximating 1:1 (Figure S1c), confirming the successful synthesis of the precursor with an Fe:Co atomic ratio of 1:1. The SEM images of commercially acquired *s*-FeCo alloy particles are presented in Figure 2a_1_,a_2_, revealing a smooth surface morphology and predominantly spherical particle shapes. The average particle size of these spherical particles was 6.8 μm. Conversely, the SEM images of *f*-FeCo alloy particles synthesized via MPARCVD technology are displayed in Figure 2c_1_,c_2_. Under a H_2_ atmosphere, the hydrogen and oxygen atoms in the *f*-Fe(OH)_3_/Co(OH)_2_ precursor were liberated, resulting in the formation of pores of varying sizes and coral-like *f*-FeCo alloy particles. As is evident from the high-magnification SEM image (Figure 2c_2_), these coral-like *f*-FeCo alloy particles were constructed by the stacking of two-dimensional alloy nanosheets, which possessed the potential to serve as catalysts for the subsequent growth of carbon materials. The particle sizes of the flake-shaped FeCo were uneven, displaying a heterogeneous particle size distribution spanning from 0.5 μm to 2.0 μm. The FESEM images of *f*-FeCo@CM are presented in Figure 2d_1_,d_2_. The high-magnification SEM image (Figure 2d_2_) demonstrated that upon the introduction of CH_4_, carbon nanoparticles covered the surface of *f*-FeCo in the form of carbon spheres, with a particle size distribution ranging from 0.1 μm to 0.3 μm. Notably, the coverage density of these carbon spheres was extremely high, resulting in the formation of a flower-like cluster of *f*-FeCo@CM composite material through the accumulation of a large number of carbon spheres (Figure 2d_1_). Additionally, the elemental mapping depicted in Figure 2c_3_,d_3_ revealed a uniform distribution of Fe and Co elements in the *f*-FeCo alloy particles and *f*-FeCo@CM composites. Notably, the C element content detected on the surface of the *f*-FeCo@CM composite significantly exceeded those of the Fe and Co elements, confirming the successful coating of numerous carbon microspheres onto the surface of the *f*-FeCo alloy. The FESEM images of *s*-FeCo@CM (Figure 2b_1_,b_2_) revealed that the carbon spheres deposited on the surface of *s*-FeCo alloy particles exhibited a broader particle size distribution, ranging from 0.1 μm to 0.7 μm. The density of these carbon spheres was significantly lower compared to that of *f*-FeCo@CM, indicating a lower coating efficiency of carbon material growth on the surface of *s*-FeCo. This observation demonstrated the distinct morphological characteristics of the carbon microspheres formed on different FeCo substrates. This was attributed to the stacked laminar structure of *f*-FeCo, which provided more exposed edges than spherical particles. These edges possessed higher catalytic activity, and the abundance of edges enabled *f*-FeCo to possess a greater number of active sites for carbon deposition [44,45,46]. Consequently, the surface of *f*-FeCo allowed for the deposition and coating of significantly more carbon microspheres than that of *s*-FeCo. The increased deposition and coating of carbon materials provided more dielectric loss components for the magnetic materials, thereby enhancing their impedance-matching performance.

**Figure 3:**
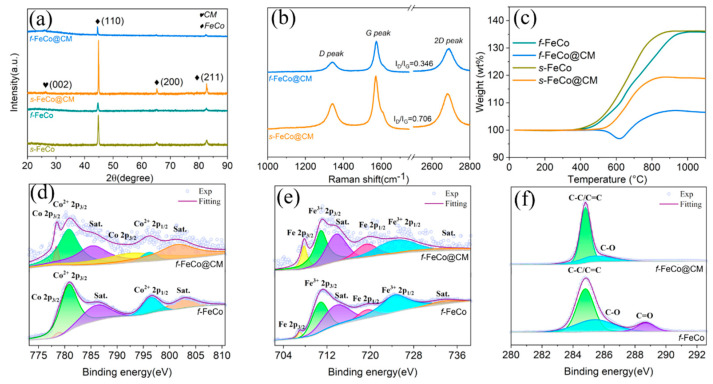
(**a**) XRD patterns of different samples; (**b**) Raman spectra of *s*-FeCo@CM and *f*-FeCo@CM; (**c**) TGA patterns of *f*-FeCo and *f*-FeCo@CM samples; XPS spectra of (**d**) Co *2p*, (**e**) Fe *2p*, and (**f**) C *1s* for *f*-FeCo and *f*-FeCo@CM samples.

The microstructural and phase compositional analyses of the samples were conducted using XRD patterns. Initially, the XRD pattern of the *f*-Fe(OH)_3_/Co(OH)_2_ precursor (Figure S2a) was examined, revealing peaks at 11°, 22.1°, and 36.8°, attributable to the (0 0 3), (0 0 6), and (1 0 1) crystal planes of the Co(OH)_2_ (JCPDS #01-0357), respectively. Additionally, characteristic peaks observed at 30.8° and 62.7° corresponded to the (1 1 1) and (4 4 0) crystal planes of the Fe(OH)_3_ (JCPDS #22-0346), thereby confirming the successful synthesis of the *f*-Fe(OH)_3_/Co(OH)_2_ precursor. Subsequently, XRD phase analysis (Figure 3a) revealed distinct peaks at 44.8°, 65.3°, and 82.7° for both *s*-FeCo and *f*-FeCo samples, which matched the (1 1 0), (2 0 0), and (2 1 1) crystal planes of the FeCo (JCPDS #44-1433). This indicated the successful preparation of *f*-FeCo metal particles utilizing the MPARCVD technique in this study. Moreover, the XRD patterns of both *s*-FeCo@CM and *f*-FeCo@CM samples exhibited a peak at 26.4°, corresponding to the (0 0 2) crystal plane of the graphitized carbon (JCPDS #01-0640). This finding confirmed the successful deposition and coating of carbon materials on the surfaces of *s*-FeCo and *f*-FeCo samples.

To further probe into the graphitization level of the deposited carbon, Raman spectroscopy was employed. As depicted in Figure 3b, both *s*-FeCo@CM and *f*-FeCo@CM samples exhibited three distinctive characteristic peaks, situated near 1350 cm^−1^, 1580 cm^−1^, and 2700 cm^−1^, corresponding to the *D*, *G*, and *2D* peaks of graphitized carbon, respectively. The *D* peak serves as an indicator of the proportion of defects, structural distortions, and amorphous regions within the samples. Meanwhile, the *G* peak represents the primary characteristic peak of graphene. The *I_D_*/*I_G_* ratio, which measures the intensity ratio between the *D* and *G* peaks, can be used to assess the graphitization degree of the carbon material. Specifically, a lower *I_D_*/*I_G_* value suggests a reduced defect density and a higher graphitization level [47,48]. Based on the analysis in Figure 3b, the *I_D_*/*I_G_* ratio for *f*-FeCo@CM was determined to be 0.348, whereas the corresponding value for *s*-FeCo@CM was 0.706. This significantly lower *I_D_/I_G_* ratio observed for *f*-FeCo@CM compared to *s*-FeCo@CM indicated that the carbon microspheres deposited onto the *f*-FeCo alloy exhibited fewer defects, a higher crystallinity, and a superior graphitization level. This finding validated the successful deposition of carbon microspheres and the coating of a graphite layer onto the surface of *f*-FeCo metal particles using the MPARCVD technique, resulting in the production of high-purity *f*-FeCo@CM composite materials. Furthermore, the quality of the deposited carbon material surpassed that coated onto the surface of *s*-FeCo. 

To gain further insights into the variation in oxidation resistance between *f*-FeCo and *f*-FeCo@CM, a chemical element analysis was performed using XPS. In the XPS survey spectrum of *f*-FeCo@CM (Figure S2b), distinct peaks corresponding to C *1s*, O *1s*, Fe *2p*, and Co *2p* were observed, indicating the presence of C, O, Fe, and Co elements in the composite. Notably, a substantial increase in the C *1s* peak intensity of *f*-FeCo@CM compared to *f*-FeCo was discerned after carbon deposition, whereas the Co *2p* and Fe *2p* peaks exhibited a dramatic reduction. This phenomenon was attributed to the coating of a significant amount of carbon material on the surface of *f*-FeCo@CM, which attenuated the Fe *2p* and Co *2p* peaks. To evaluate the valence states and bonding states of each element, high-resolution spectra were further analyzed. The Co *2p* spectrum (Figure 3d) revealed six distinct peaks, with Co *2p*_3/2_ detected at 778.7 eV and 793.5 eV, indicating the presence of zerovalent cobalt. Peaks corresponding to Co^2+^ *2p*_3/2_ and its satellite peak were observed at 781.2 eV and 786.0 eV, while Co^2+^ *2p*_1/2_ and its satellite peak were detected at 797.3 eV and 802.8 eV, both representative of divalent cobalt. The Fe *2p* spectrum (Figure 3e) displayed six small peaks, comprising Fe *2p*_3/2_ characteristic peaks and their satellite peaks at 707.5 eV and 713.7 eV, and Fe *2p*_1/2_ detected at 719.0 eV, all attributed to zerovalent iron. Additionally, Fe^3+^ *2p*_3/2_ was identified at 710.9 eV, and the peak of Fe^3+^ *2p*_1/2_ and its satellite peak were observed at 724.8 eV and 733.4 eV, both indicative of trivalent iron. The presence of divalent cobalt and trivalent iron was attributed to the inevitable oxidation process that occurs on the surface of *f*-FeCo. Furthermore, the deconvoluted areas of Co^2+^ *2p*_3/2_, Co^2+^ *2p*_1/2_, Fe^3+^ *2p*_3/2_, and Fe^3+^ *2p*_1/2_ in the XPS spectra of *f*-FeCo@CM were obviously smaller than those of *f*-FeCo, while the deconvoluted areas of Co *2p*_3/2_ and Fe *2p*_3/2_ were significantly larger. This observation suggested that the carbon microspheres coating on the surface of *f*-FeCo@CM shielded the inner *f*-FeCo effectively, significantly improving the oxidation resistance. In the C *1s* spectrum (Figure 3f), three distinct peaks were observed. The peaks corresponding to C-C/C=C bonds (representing the bonding state of graphitic carbon) were detected at 284.8 eV and 285.3 eV, while C-O bonds and C=O bonds were identified at 288.7 eV. Notably, the deconvoluted area of the C-O bond peak in f-FeCo@CM was reduced, and the characteristic peak of the C=O bond disappeared, indicating a decrease in oxidized carbon species. In contrast, the deconvoluted area of the C-C/C=C bond was significantly larger than that of *f*-FeCo, which further validated that carbon deposition could significantly enhance the oxidation resistance of *f*-FeCo alloy particles.

To assess the relative composition content and antioxidant capacity of different samples, TG analysis was performed in an air atmosphere (Figure 3c). The *s*-FeCo commenced weight gain at 248 °C, reaching 136% and remaining stable at 907 °C. Similarly, the *f*-FeCo initiated weight gain at 295 °C, achieving 136% and maintaining it until 942 °C. The substantial weight increase in both samples was predominantly attributed to the oxidation of FeCo species, reflecting their relatively low antioxidant capacity. In contrast, the *s*-FeCo@CM sample started gaining weight at 443 °C, peaking at 119% and remaining stable at 860 °C. Interestingly, the *f*-FeCo@CM sample exhibited a change in weight only at 500 °C, differing from *s*-FeCo@CM by initially decreasing to 96% at 620 °C, followed by an increase to 107% at 893 °C. The weight gain in both *s*-FeCo@CM and *f*-FeCo@CM samples was due to the oxidation of FeCo species, while the weight loss in *f*-FeCo@CM was attributed to the combustion of carbon species. Notably, the initial change temperatures for both samples were significantly higher than those of the FeCo samples, indicating that the deposition of carbon material on the FeCo surface effectively enhanced the antioxidant properties. Furthermore, the temperature at which the *f*-FeCo@CM sample began to exhibit weight changes was higher than that of *s*-FeCo@CM, suggesting that the high-density carbon coating of *f*-FeCo@CM resulted in a more significant improvement in antioxidant performance. Furthermore, only the *f*-FeCo@CM sample exhibited a significant weight reduction due to carbon combustion, indicating a higher carbon deposition content compared to *s*-FeCo, which aligned with FESEM observations. Additionally, calculations revealed that the *s*-FeCo@CM contained 88% *s*-FeCo species and 12% carbon species, while the *f*-FeCo@CM comprised 78% *f*-FeCo species and 22% carbon species.

**Figure 4:**
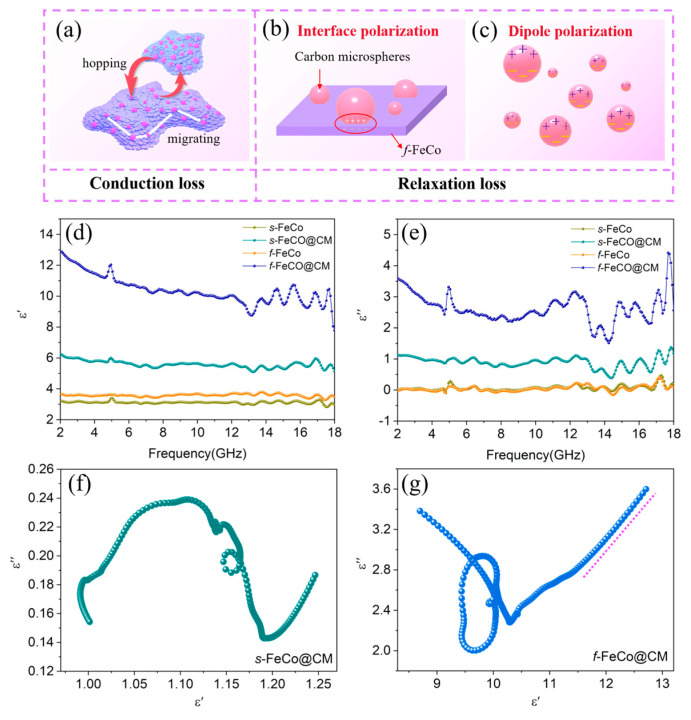
(**a**–**c**) Schematic diagrams illustrating the dielectric loss mechanism of *f*-FeCo@CM; (**d**,**e**) real and imaginary parts of the permittivity values for different samples; (**f**) *ε*′–*ε*″ curve of *s*-FeCo@CM and (**g**) *ε*′–*ε*″ curve of *f*-FeCo@CM.

The electromagnetic parameters of various samples, encompassing the complex permittivity (*ε_r_* = *ε*′ − j*ε*″) and complex permeability (*μ_r_* = *μ*′ − j*μ*″), were employed to elucidate the influence of composition and microstructural characteristics on microwave absorption properties. Specifically, the real parts (*ε*′ and *μ*′) signify the storage capacity of electric and magnetic energy, while the imaginary parts (*ε*″ and *μ*″) reflect the dissipation capability of these energies. As depicted in Figure 4d,e, the *ε*″ values of *s*-FeCo and *f*-FeCo were apparently small, remaining below 1.0 across the entire measurement spectrum. However, following the deposition of carbon microspheres, both *ε*′ and *ε*″ values underwent a significant increase. Precisely, the *ε*′ values of *s*-FeCo@CM and *f*-FeCo@CM spanned ranges from 12.90 to 7.82 and 6.25 to 5.05, respectively, whereas the *ε*″ values of these samples ranged from 4.42 to 1.51 and 1.38 to 0.38, respectively. This marked augmentation underscored the prominent enhancement in the dielectric loss capacity achieved through the integration of FeCo and carbon materials. Additionally, the *ε*′ and *ε*″ values of *f*-FeCo@CM were substantially higher than those of *s*-FeCo@CM, with the *ε*′ value of *f*-FeCo also being significantly elevated compared to *s*-FeCo, which suggested that the *f*-FeCo exhibited superior carbon deposition catalysis due to its lamellar structure, resulting in a denser and more homogeneous carbon coating. This optimal configuration allowed *f*-FeCo@CM to possess superior dielectric loss performance. Furthermore, the presence of multiple resonance peaks in the *ε*″ value of *f*-FeCo@CM indicated the existence of diverse dielectric loss mechanisms. Based on the permittivity curves, the tangent of the dielectric loss (tan*δ_ε_* = *ε*″/*ε*′) was calculated (seen in Figure S3c) to assess the dielectric loss capabilities. As observed, the tan*δ_ε_* values of *f*-FeCo and *s*-FeCo were exceptionally low. However, following the deposition of carbon, both samples exhibited a substantial increase in tan*δ_ε_* values. Moreover, the tan*δ_ε_* value of *f*-FeCo@CM was significantly higher than that of *s*-FeCo@CM, which confirmed that the coating of carbon microspheres effectively enhanced the dielectric loss capability, and the deposition of carbon microspheres on *f*-FeCo synthesized via MPARCVD technology further improved the dielectric loss performance of *f*-FeCo@CM, in alignment with the findings derived from the permittivity analysis. 

Typically, dielectric loss is attributed to conduction loss and polarization loss. Conduction loss is inherently linked to conductivity, whereas polarization loss depends on interfacial polarization and dipole polarization [49]. The Debye theory [50] is employed to analyze dielectric loss and can be described by the following equations:

(3) $$\varepsilon^{\prime }=\varepsilon_{\infty }+\frac{\varepsilon_{s}-\varepsilon_{\infty }}{1+\omega^{2}\tau^{2}}$$

(4) $$\varepsilon^{\prime \prime }=\frac{\varepsilon_{s}-\varepsilon_{\infty }}{1+\omega^{2}\tau^{2}}\omega \tau +\frac{\sigma }{\omega \varepsilon_{0}}$$

(5) $${\left(\varepsilon^{\prime }-\frac{\varepsilon_{s}+\varepsilon_{\infty }}{2}\right)}^{2}+{\left(\varepsilon^{\prime \prime }\right)}^{2}={\left(\frac{\varepsilon_{s}+\varepsilon_{\infty }}{2}\right)}^{2}$$

wherein *ε_s_* represents the static permittivity, *ε_∞_* represents the optical permittivity, *ω* is the angular frequency, *τ* is the relaxation time, and *σ* is the conductivity. The relationship between *ε*′ and *ε*″ is expressed in Equation (5). When the *ε*′–*ε*″ curve forms a semicircle, referred to as the Cole–Cole semicircle, it signifies a polarization relaxation process. Conversely, the presence of a straight-line segment indicates the occurrence of a conductive loss process [51]. Figure 4g depicts the *ε*′–*ε*″ curve of the *f*-FeCo@CM sample, which exhibited a characteristic morphology frequently encountered in studies pertaining to the development and performance assessment of low-frequency absorbing materials [52,53,54]. Notably, multiple distinct semicircles were discernible within the range of 8.0–11.5 for *ε*′, corresponding to the 4–18 GHz frequency band depicted in Figure 4d. This observation indicated the presence of multiple polarization relaxation processes occurring in the *C*, *X*, and *Ku* band, which signified that polarization loss was the predominant loss mechanism within these frequency ranges. Furthermore, a straight-line segment emerged at the tail of the *ε*′–*ε*″ curve, spanning the range of 11.5–13.0 for *ε*′. This segment corresponded to the 2–4 GHz frequency band shown in Figure 4d, indicating that in the *S* band, the loss mechanism shifted primarily towards conductive loss. This finding demonstrated a combination of polarization losses and conductive loss across the broad frequency range of 2–18 GHz for *f*-FeCo@CM. As depicted in Figure 4b, the *f*-FeCo@CM exhibited numerous heterogeneous interfaces between carbon microspheres and *f*-FeCo, which triggered current hysteresis displacement and an uneven distribution of positive and negative charges under alternating electric fields, which facilitated the generation of spatial electric dipole moments, ultimately inducing abundant interface polarization. Under the application of an external electromagnetic field, dipole pairs concentrated at the heterogeneous interfaces may become sources of dipole polarization (Figure 4c). As shown in Figure 4a, the carbon microspheres deposited on the surface of *f*-FeCo provided an ample supply of free electrons, enabling their movement on the *f*-FeCo alloy sheets and jumps between alloy particles. Consequently, an efficient conductive network was established within the matrix, leading to an enhancement in conductivity. Conversely, the *ε*′–*ε*″ curve of *s*-FeCo@CM (Figure 4f) solely exhibited semicircles, and was devoid of straight-line segments, suggesting that the loss mechanism was exclusively attributed to polarization relaxation loss, excluding conductive loss. Furthermore, the *ε*′–*ε*″ curves of *s*-FeCo and *f*-FeCo, as depicted in Figure S3a,b, did not exhibit distinct semicircles or straight-line segments, indicating that the loss process in FeCo alloys hardly involved polarization relaxation loss or conductive loss under a relatively low loading content, resulting in significantly lower dielectric loss intensity compared to materials compounded with carbon materials.

**Figure 5:**
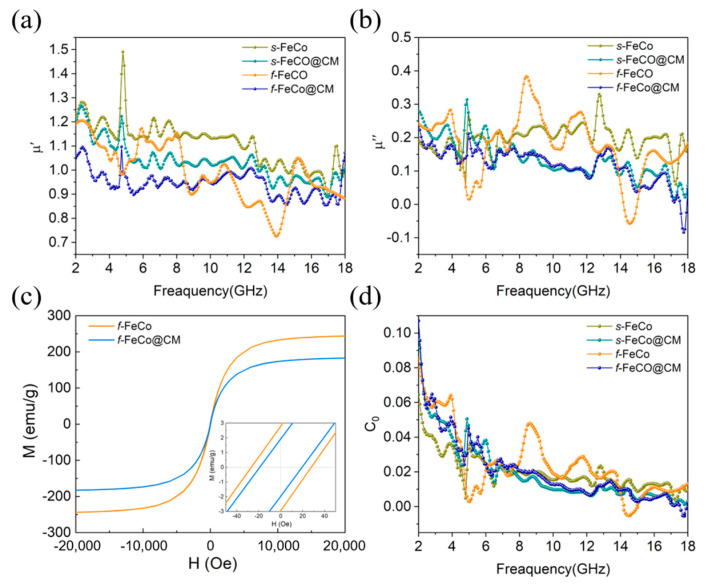
(**a**,**b**) The real and imaginary parts of the permeability values for different samples; (**c**) hysteresis loops of *f*-FeCo and *f*-FeCo@CM; (**d**) the eddy–current modeling plots.

The variation of *μ*′ and *μ*″ values with frequency for various samples is depicted in Figure 5a,b. The *f*-FeCo sample exhibited *μ*′ and *μ*″ values fluctuating within the ranges of 1.29–0.70 and 0.73–0.03, respectively. In the case of *f*-FeCo@CM, the *μ*′ and *μ*″ values varied between 1.07–0.85 and 0.24–0, respectively. The *μ*′ and *μ*″ values for *s*-FeCo ranged from 1.49–0.91 and 0.33–0.05, while those of *s*-FeCo@CM were within the ranges of 1.27–0.89 and 0.31–0.02, respectively. In contrast to the dielectric constant curves of these four samples, the differences in *μ*′ and *μ*″ values were minimal, indicating that the introduction of carbon materials had a limiting effect on the magnetic properties of the FeCo alloy. This observation was attributed to the excellent chemical stability of carbon microspheres, which effectively enhanced the oxidation resistance of the magnetic metals, thereby minimizing structural damage and preserving their inherent magnetic properties. The magnetization curves (*M*-*H*) of the four samples were measured at room temperature, as shown in Figure 5c,d. According to the literature [55], the *μ_r_* can be calculated using the following equation:

(6) $$\mu_{r}=\frac{M_{s}^{2}}{\mu_{0}\cdot (\alpha \cdot \kappa \cdot H_{c}\cdot M_{s}+b\lambda \xi )}$$

where α and b are constants determined by the material composition, *κ* is the proportionality coefficient, *λ* is the magnetostrictive coefficient, and *ξ* is the elastic strain parameter. As can be seen from the above equation, a higher magnetic saturation (*M_s_*) value and a lower coercivity (*H_c_*) value were found to enhance the permeability (*μ_r_*) of the material, thereby indicating improved magnetic loss capability. As displayed in Figure 5c, the *M_s_* values for *f*-FeCo and *f*-FeCo@CM were recorded as 243.9 emu/g and 182.8 emu/g, respectively, while the *H_c_* values were 28.1 Oe and 19.1 Oe, correspondingly. Notably, the *M_s_* and *H_c_* values of *f*-FeCo@CM exhibited a slight reduction compared to *f*-FeCo, as is evident from the bar chart depicted in Figure S4b. The *M_s_* value was influenced by the atomic magnetic moment and the density of ferromagnetic atoms per unit volume [56]. The deposition of non-ferromagnetic carbon microspheres could reduce the number of ferromagnetic atoms per unit volume, thus leading to a decrease in the *M_s_* value. Furthermore, the coercivity of magnetic materials was closely linked to magnetic crystal anisotropy and material defects [57]. Given the similarity in composition and morphology of *f*-FeCo alloy, the variations in *H_c_* in this study were dependent on the defect conditions within the material system. According to the magnetic domain wall pinning model [58], disordered defects within the material impede the movement of magnetic domain walls, resulting in elevated *H_c_* values. However, in this study, the deposition of graphitized carbon microspheres mitigated the disordered defects on the surface of *f*-FeCo alloy particles, thereby resulting in a lower *H_c_* value for *f*-FeCo@CM compared to *f*-FeCo. Upon thorough analysis, it was discerned that the magnetic properties of *f*-FeCo were less influenced after being coated with carbon microspheres, aligning with the permeability analysis results. Further examination of the magnetic loss tangent (tan*δ_μ_* = *μ*″/*μ*′) for the four samples, as depicted in Figure S3c, revealed the presence of multiple resonance peaks. These peaks were attributed to surface effects, nano-size effects, and spin-wave excitations [59]. In the context of magnetic theory, the magnetic loss was intricately linked to domain wall resonance, hysteresis loss, natural resonance, exchange resonance, and eddy current loss. For the microwave absorption band spanning 2–18 GHz, domain wall resonance and hysteresis loss can be disregarded [60]. According to Aharoni’s theory [61], the resonance peaks observed in the 2–8 GHz range of the tan*δ_μ_* curve were attributed to natural resonance, while those in the higher frequency range correspond to exchange resonance. Additionally, eddy current effects constituted a significant contributor to magnetic loss [62]. To gain a deeper understanding of the role of eddy current loss, the *C*_0_ coefficient was introduced and calculated by the following equation:

(7) $$C_{0}=\mu^{\prime \prime }{\left(\mu^{\prime }\right)}^{-2}f^{-1}$$

According to commonly accepted standards, the eddy current loss is considered a component of magnetic loss when the *C*_0_ value remains constant with increasing frequency. Conversely, if the *C*_0_ value is invariant across frequencies, the impact of eddy current loss is negligible. As depicted in Figure 5d, the eddy current curves of all four samples underwent substantial variations with frequency, thereby excluding the influence of eddy current loss on the magnetic loss. This observation suggested that the magnetic loss in *s*-FeCo@CM and *f*-FeCo@CM composites was primarily attributed to natural resonance and exchange resonance, stemming from the intrinsic magnetic properties of *s*-FeCo and *f*-FeCo.

**Figure 6:**
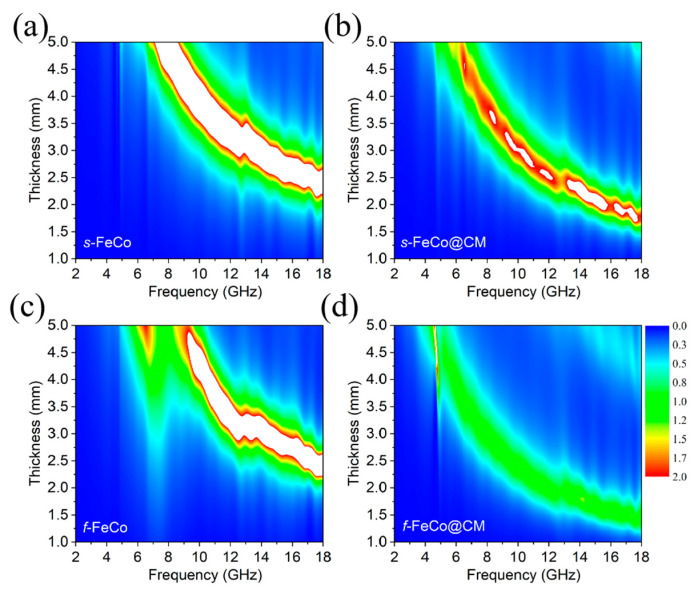
Two-dimensional representations of the *Z*-value for different samples: (**a**) *s*-FeCo; (**b**) *s*-FeCo@CM; (**c**) *f*-FeCo; (**d**) *f*-FeCo@CM.

The impedance matching characteristics and attenuation constant are significant factors determining the absorption performance of EAMs. The impedance matching characteristics can be evaluated using the following formula [63]:

(8) $$Z_{in}=Z_{0}\sqrt{\left(\frac{\mu_{r}}{\varepsilon_{r}}\right)}\tan h\left\{j\left(\frac{2\pi fd}{c}\right)\sqrt{\mu_{r}\varepsilon_{r}}\right\}$$

(9) $$|Z|=\left|\frac{Z_{in}}{Z_{0}}\right|$$

wherein *Z*_0_ and *Z_in_* represent the impedance of free space and the input impedance of the electromagnetic wave absorber, respectively, *ε_r_* and *μ_r_* are the complex permittivity and complex permeability, respectively, *f* is the frequency of the electromagnetic wave, *d* is the thickness of the electromagnetic wave absorber, and *c* is the speed of light. Good impedance matching typically requires that the absolute value of the impedance (|*Z*|) approximates or equals 1. The |*Z*| values of the four samples are presented in 2D color maps in Figure 6a–d, where green areas signify the proximity of |*Z*| to 1. Upon introducing carbon materials into the magnetic material system, whether in the form of *s*-FeCo@CM or *f*-FeCo@CM, a remarkable expansion in the green regions of their respective 2D color maps can be observed, indicating a significant enhancement in impedance matching characteristics. Specifically, *f*-FeCo@CM (depicted in Figure 6d) exhibited the most optimal impedance matching characteristic, suggesting that the utilization of lamellar *f*-FeCo alloy as a catalyst for growing carbon microspheres on its surface could enhance the dielectric properties of the composite, thus attaining the best impedance matching performance. The attenuation constant serves as a crucial parameter for evaluating the capacity of EAMs to attenuate electromagnetic waves, where a higher value indicates a stronger ability to absorb and convert electromagnetic wave energy. The attenuation constant (*α*) was calculated using the following formula [64]:

(10) $$\alpha =\frac{\sqrt{2\pi f}}{c}\times \sqrt{\left(\mu^{\prime \prime }\varepsilon^{\prime }-\mu^{\prime }\varepsilon^{\prime \prime }\right)+\sqrt{{\left(\mu^{\prime \prime }\varepsilon^{\prime \prime }-\mu^{\prime }\varepsilon^{\prime }\right)}^{2}-{\left(\mu^{\prime }\varepsilon^{\prime \prime }+\mu^{\prime \prime }\varepsilon^{\prime }\right)}^{2}}}$$

**Figure 7:**
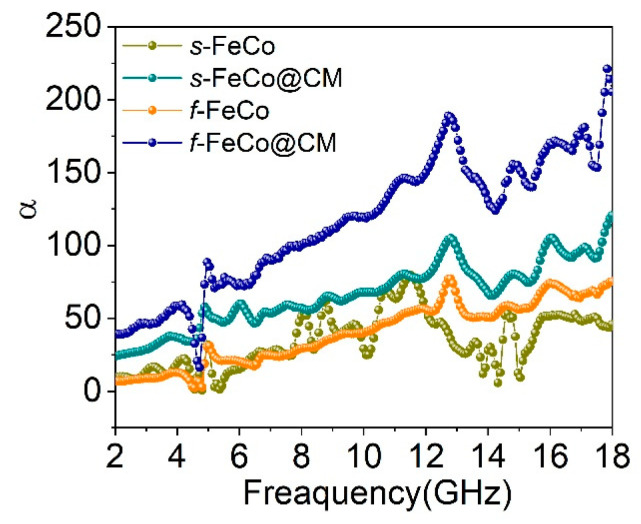
Plots of the attenuation constant (*α*) as a function of frequency for different samples.

The correlations between the *α* values and frequency for the four samples are displayed in Figure 7. Initially, it was evident that following the integration of carbon microspheres into the magnetic material system, the *α* values of both *s*-FeCo@CM and *f*-FeCo@CM underwent an increase, although the enhancement in α for *f*-FeCo@CM was more significant than that observed for *s*-FeCo@CM. This augmentation was attributed to the superior quality and quantity of carbon microspheres deposited onto the surface of *f*-FeCo. Specifically, *f*-FeCo@CM exhibited the highest α value, peaking at 221, indicative of its superior electromagnetic wave attenuation capabilities. Furthermore, as the frequency escalated, an upward trend in the α values of all samples was observed. Consequently, the growth of graphitized carbon microspheres on the surface of lamellar *f*-FeCo alloy was found to concurrently optimize the impedance matching and electromagnetic wave attenuation performance of the magnetic FeCo alloy. 

**Figure 8:**
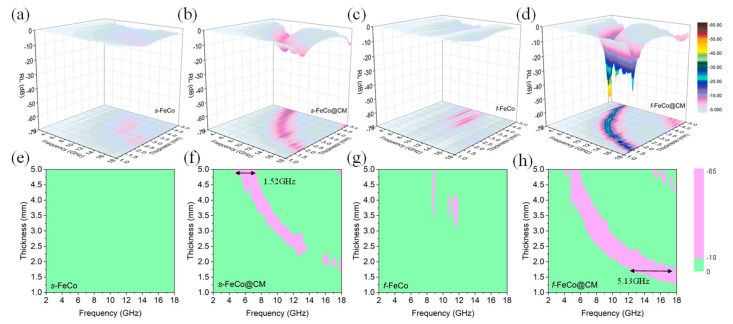
Three-dimensional color maps of reflection loss (RL) for different samples: (**a**) *s*-FeCo; (**b**) *s*-FeCo@CM; (**c**) *f*-FeCo; (**d**) *f*-FeCo@CM; two-dimensional color maps of reflection loss (RL) for different samples: (**e**) *s*-FeCo; (**f**) *s*-FeCo@CM; (**g**) *f*-FeCo; (**h**) *f*-FeCo@CM.

The RL values for four different samples were calculated, and their respective 3D color maps representing the frequency and thickness dependences are depicted in Figure 8a–d, while the corresponding 2D color maps are presented in Figure 8e–h. Upon growing carbon microspheres on the surfaces of FeCo magnetic alloy metals, the performances of both the RL and the *f*_E_ improved significantly. Specifically, *s*-FeCo attained an RL_min_ value of −7.1 dB at 12.8 GHz with a matching thickness of 3.5 mm. In contrast, *s*-FeCo@CM achieved an RL_min_ value of −15.1 dB at 6 GHz, accompanied by a matching thickness of 5 mm and an *f*_E_ of 1.52 GHz (5.68–7.20 GHz). For *f*-FeCo, an RL_min_ value of −12.8 dB was recorded at 8.8 GHz, with a matching thickness of 4.4 mm and an *f*_E_ of 0.16 GHz (8.72–8.88 GHz). However, *f*-FeCo@CM demonstrated a remarkable RL_min_ value of −58.2 dB at 7.84 GHz, accompanied by a significantly reduced matching thickness of only 3.0 mm and an expanded *f*_E_ of 5.13 GHz (12.31–17.44 GHz). This exceptional reflection loss performance of *f*-FeCo@CM was attributed to the composite structure of lamellar *f*-FeCo alloy and carbon microspheres, which concurrently enhanced its impedance matching and electromagnetic wave dissipation capabilities, thus making it a promising candidate for achieving high-strength EAM. 

## 4. Conclusions

In summary, the *f*-FeCo@CM composite was successfully synthesized via an advanced MPARCVD technique; the lamellar *f*-FeCo particles were efficiently decorated with a substantial amount of high-quality graphitized carbon microspheres. This approach introduced numerous heterogeneous interfaces, leading to the creation of spatial electric dipole moments and triggering robust interface polarization. Additionally, the carbon microspheres imparted a considerable number of free electrons, thereby augmenting conduction loss. Consequently, the dielectric loss of the *f*-FeCo@CM composite primarily stemmed from interface and dipole polarization, along with conduction loss. The magnetic loss of *f*-FeCo@CM was primarily attributed to natural resonance and exchange resonance, stemming from the inherent magnetic properties of *f*-FeCo, which remained largely unaffected following carbon deposition. This allowed for effective modulation of the permittivity value of *f*-FeCo@CM without compromising its magnetic loss capabilities, significantly enhancing the dielectric loss capacity and further optimizing impedance matching. Notably, *f*-FeCo@CM exhibited exceptional electromagnetic wave absorption performance at a 40 wt% filling content. Specifically, at a frequency of 7.84 GHz, it achieved an RL_min_ of −58.2 dB and an *f*_E_ with a span of 5.13 GHz (12.31–17.44 GHz). In contrast, the *s*-FeCo@CM composite synthesized under identical conditions displayed a significantly lower RL_min_ of −15.1 dB and a narrower *f*_E_ span of 1.52 GHz (5.68–7.2 GHz). This demonstrates that the flaky *f*-FeCo synthesized through the combined hydrothermal synthesis and MPARCVD techniques in this study possessed a superior electromagnetic wave absorption potential, positioning *f*-FeCo@CM composites as promising candidates for advanced EAMs in various future applications. 

## Data Availability

Data are contained within the article.

## Supplementary Materials

The following supporting information can be downloaded at https://www.mdpi.com/article/10.3390/nano14141194/s1: Figure S1: (a,b) SEM images and (c) EDS scanning element distribution map of Fe(OH)_3_/Co(OH)_2_ precursor. Figure S2: (a) XRD pattern of *f*-Fe(OH)_3_/Co(OH)_2_ precursor; (b) XPS spectra scan of *f*-FeCo and *f*-FeCo@CM samples. Figure S3: (a) *ε*′–*ε*′′ curves of *s*-FeCo and (b) *f*-FeCo; (c) dielectric loss tangent curves of different samples. Figure S4: (a) Tangent curves of magnetic loss for different samples; (b) bar chart of saturation magnetization (*M_s_*) and coercivity (*H_c_*) for *f*-FeCo and *f*-FeCo@CM samples.

## References

[1] Yang Y., Han M., Liu W., Wu N., Liu J. (2022). Hydrogel-based composites beyond the porous architectures for electromagnetic interference shielding. Nano Res..

[2] Wang Q., Niu B., Han Y., Zheng Q., Li L., Cao M. (2023). Nature-inspired 3D hierarchical structured “vine” for efficient microwave attenuation and electromagnetic energy conversion device. Chem. Eng. J..

[3] Wang X., Zhang F., Hu F., Li Y., Chen Y., Wang H., Min Z., Zhang R. (2022). N-doped honeycomb-like Ag@N-Ti_3_C_2_T_x_ foam for electromagnetic interference shielding. Nanomaterials.

[4] Li K., Han L., Wang X., Gao F., Zhang J., Cheng J. (2023). MOF-derived CoNC@rGO/amine-rich@rGO/fluorinated-epoxy nanocomposites with EMI shielding, mechanical robustness, superamphiphobicity and long-term anticorrosion properties. Chem. Eng. J..

[5] Liu H., Cui G., Li L., Zhang Z., Lv X., Wang X. (2020). Polypyrrole chains decorated on CoS spheres: A core-shell like heterostructure for high-performance microwave absorption. Nanomaterials.

[6] Ma M., Zheng Q., Zhang X., Li L., Cao M. (2023). VSe_2_/CNTs nanocomposites toward superior electromagnetic wave absorption performance. Carbon.

[7] Yang X., Qin Y., Peng L., Pan M., Xu H. (2023). Lightweight and anti-corrosive carbon nanotubes (CNTs)/bamboo fiber/HDPE composite for efficient electromagnetic interference shielding. Colloid Surf. A.

[8] Liao Y., Yi E., Zhou X., He G. (2022). Quantum dots with Mott-Schottky effect embedded in crystal-amorphous carbon for broadband electromagnetic wave absorption. J. Alloys Compd..

[9] Che R., Peng L., Duan X., Chen Q., Liang X. (2004). Microwave absorption enhancement and complex permittivity and permeability of Fe encapsulated within carbon nanotubes. Adv. Mater..

[10] Lv H., Yang Z., Wang P.L., Ji G., Song J., Zheng L., Xu Z.J. (2018). A voltage-boosting strategy enabling a low-frequency, flexible electromagnetic wave absorption device. Adv. Mater..

[11] Liu Q., Cao Q., Bi H., Liang C., Yuan K., She W., Yang Y., Che R. (2016). CoNi@SiO_2_@TiO_2_ and CoNi@ Air@TiO_2_ microspheres with strong wideband microwave absorption. Adv. Mater..

[12] Cao M., Wang X., Cao W., Fang X., Wen B., Yuan J. (2018). Thermally driven transport and relaxation switching self-powered electromagnetic energy conversion. Small.

[13] Sista K., Dwarapudi S., Kumar D., Sinha G., Moon A. (2021). Carbonyl iron powders as absorption material for microwave interference shielding: A review. J. Alloys Compd..

[14] Cheng J., Zhang H., Ning M., Raza H., Zhang D., Zheng G., Zheng Q., Che R. (2022). Emerging materials and designs for low- and multi-band electromagnetic wave absorbers: The search for dielectric and magnetic synergy. Adv. Funct. Mater..

[15] Zhang Y., Kong J., Gu J. (2022). New generation electromagnetic materials: Harvesting instead of dissipation solo. Sci. Bull..

[16] Liang B., Wang S., Kuang D. (2018). Facile synthesis and excellent microwave absorption properties of FeCo-C core–shell nanoparticles. Nanotechnology.

[17] Lv H., Ji G., Wang M. (2014). Hexagonal-cone like of Fe_50_Co_50_ with broad frequency microwave absorption, Effect of ultrasonic irradiation time. J. Alloys Compd..

[18] Chen N., Jiang J., Xu C. (2017). Co_7_Fe_3_ and Co_7_Fe_3_@SiO_2_ nanospheres with tunable diameters for high-performance electromagnetic wave absorption. ACS Appl. Mater. Interfaces..

[19] Kuang D., Hou L., Wang S. (2019). Large-scale synthesis and outstanding microwave absorption properties of carbon nanotubes coated by extremely small FeCo-C core-shell nanoparticles. Carbon.

[20] Jiang Q., Liu H., Cao Z. (2017). Synthesis and enhanced electromagnetic wave absorption performance of amorphous Co_x_Fe_10−x_ alloys. J. Alloys Compd..

[21] Cheng Y., Ji G., Li Z. (2017). Facile synthesis of FeCo alloys with excellent microwave absorption in the whole Ku-band, Effect of Fe/Co atomic ratio. J. Alloys Compd..

[22] Yang Z., You W., Xiong X. (2022). Morphology-evolved succulent-like FeCo microarchitectures with magnetic configuration regulation for enhanced microwave absorption. ACS Appl. Mater. Interfaces.

[23] Wang Y., Sun Y., Zong Y. (2020). Carbon nanofibers supported by FeCo nanocrystals as difunctional magnetic/dielectric composites with broadband microwave absorption performance. J. Alloys Compd..

[24] Wang B., Ding M., Shao C. (2023). Facile synthesis of Co_x_Fe_y_@C nanocomposite fibers derived from pyrolysis of cobalt/iron chelate nanowires for strong broadband electromagnetic wave absorption. Chem. Eng. J..

[25] Ajia S., Asa H., Toyoda Y. (2022). Development of an alternative approach for electromagnetic wave absorbers using Fe–Cr–Co alloy powders. J. Alloys Compd..

[26] Zhu H., Jiao Q., Fu R. (2022). Cu/NC@Co/NC composites derived from core-shell Cu-MOF@Co-MOF and their electromagnetic wave absorption properties. J. Colloid Interf. Sci..

[27] Amini M., Kamkar M., Rahmani F. (2021). Multilayer structures of a Zn_0.5_Ni_0.5_Fe_2_O_4_-reduced graphene oxide/PVDF nanocomposite for tunable and highly efficient microwave absorbers. ACS Appl. Electron. Mater..

[28] Wen C., Xiao L., Zhang R. (2021). High-density anisotropy magnetism enhanced microwave absorption performance in Ti_3_C_2_T_x_ MXene@Ni microspheres. ACS Nano.

[29] Qiang R., Du Y., Zhao H. (2015). Metal organic framework-derived Fe/C nanocubes toward efficient microwave absorption. J. Mater. Chem. A.

[30] Lu M., Cao M., Chen Y., Cao W., Liu J., Shi H., Zhang D., Wang W., Yuan J. (2015). Multiscale assembly of grape-like ferroferric oxide and carbon nanotubes: A smart absorber prototype varying temperature to tune intensities. ACS Appl. Mater. Interfaces.

[31] Chen X., Wang W., Shi T., Wu G., Lu Y. (2020). One pot green synthesis and EM wave absorption performance of MoS_2_@nitrogen doped carbon hybrid decorated with ultrasmall cobalt ferrite nanoparticles. Carbon.

[32] Zhang N., Huang Y., Liu D., Wang M. (2018). High efficiency microwave absorption nanocomposites of multiple-phase core-shell CoNi alloy@C loaded on rGO conducting network. Compos. Part A-Appl. S..

[33] Li H., Hou Y., Li L. (2019). Synthesis of the SiO_2_@C composites with high-performance electromagnetic wave absorption. Powder Technol..

[34] Liu D., Qiang R., Du Y. (2018). Prussian blue analogues derived magnetic FeCo alloy/carbon composites with tunable chemical composition and enhanced microwave absorption. J. Colloid Interf. Sci..

[35] Fan Y., Li Y., Yao Y. (2020). Hierarchically porous carbon sheets/Co nanofibers derived from corncobs for enhanced microwave absorbing properties. Appl. Surf. Sci..

[36] Li J., Miao P., Chen K. (2020). Highly effective electromagnetic wave absorbing Prismatic Co/C nanocomposites derived from cubic metal-organic framework. Compos. Part B-Eng..

[37] Wang L., Wen B., Yang H. (2020). Hierarchical nest-like structure of Co/Fe MOF derived CoFe@C composite as wide-bandwidth microwave absorber. Compos. Part A-Appl. Sci..

[38] Wang F., Wang N., Han X. (2019). Core-shell FeCo@carbon nanoparticles encapsulated in polydopamine-derived carbon nanocages for efficient microwave absorption. Carbon.

[39] Duan Y., Hu T., Yang L. (2019). Facile fabrication of electroactive microporous Co_3_O_4_ through microwave plasma etching for supercapacitors. J. Alloys Compd..

[40] Nagaraju N., Fonseca A., Konya Z., Nagy J. (2002). Alumina and silica supported metal catalysts for the production of carbon nanotubes. J. Mol. Catal. A-Chem..

[41] Willems I., Kónya Z., Colomer J., Tendeloo G., Nagaraju N., Fonseca A., Nagy J. (2000). Control of the outer diameter of thin carbon nanotubes synthesized by catalytic decomposition of hydrocarbons. Chem. Phys. Lett..

[42] Kónya Z., Kiss J., Oszkó A., Siska A., Kiricsi I. (2001). XPS characterization of catalysts during production of multiwalled carbon nanotubes. Phys. Chem. Chem. Phys..

[43] Wen J., Chu W., Jiang C., Tong D. (2010). Growth of carbon nanotubes on the novel FeCo-Al_2_O_3_ catalyst prepared by ultrasonic coprecipitation. J. Nat. Gas Chem..

[44] Liu M., Yu F., Ma C., Xue X., Fu H., Yuan H., Yang S., Wang G., Guo X., Zhang L. (2019). Effective Oxygen reduction reaction performance of FeCo alloys in situ anchored on nitrogen-doped carbon by the microwave-assistant carbon bath method and subsequent plasma etching. Nanomaterials.

[45] Yi Q., Zhang F., Song Y., Wang X., Zhang H., Li C., Piao M. (2023). One-step synthesis of cobalt nanosheets depositing with carbon microsphere by microwave plasma assisted reduction chemical vapor deposition technique against electromagnetic pollution. Carbon.

[46] Shen A., Zou Y., Wang Q., Dryfe R.A.W., Huang X., Dou S., Dai L., Wang S. (2014). Oxygen reduction reaction in a droplet on graphite, direct evidence that the edge is more active than the basal plane. Angew. Chem. Int. Edit..

[47] Sivkov D., Petrova O., Mingaleva A., Ob’edkov A., Kaverin B., Gusev S., Vilkov I. (2020). The structure and chemical composition of the Cr and Fe pyrolytic coatings on the MWCNTs’ surface according to NEXAFS and XPS Spectroscopy. Nanomaterials.

[48] Ma Y., Ma Y., Ding X., Liu Q., Pang Y., Cao Y., Zhang T. (2022). Safety assessment of graphene oxide and microcystin-LR complex, a toxicological scenario beyond physical mixture. Part. Fibre Toxicol..

[49] Zhi D., Li T., Li J., Ren H., Meng F. (2021). A review of three-dimensional graphene-based aerogels, synthesis, structure and application for microwave absorption. Compos. Part B-Eng..

[50] Zhang D., Wang H., Cheng J., Han C., Yang X., Xu J. (2020). Conductive WS_2_-NS/CNTs hybrids based 3D ultra-thin mesh electromagnetic wave absorbers with excellent absorption performance. Appl. Surf. Sci..

[51] Zhou J., Wei B., Wang M., Yao Z., Chen P., Zhou C., Li Z. (2021). Three dimensional flowerlike ZnFe_2_O_4_ ferrite loaded graphene, enhancing microwave absorption performance by constructing microcircuits. J. Alloys Compd..

[52] Zhou J., Guo F., Luo J., Hao G., Liu G., Hu Y., Zhang G., Guo H., Zhou H., Jiang W. (2022). Designed 3D heterostructure with 0D/1D/2D hierarchy for low-frequency microwave absorption in the S-band. J. Mater. Chem. C.

[53] Liu C., Liu S., Feng X., Zhu K., Lin G., Bai Z., Wang L., Liu X. (2023). Phthalocyanine-mediated interfacial self-assembly of magnetic graphene nanocomposites toward low-frequency electromagnetic wave absorption. Chen. Eng. J..

[54] Cui C., Geng L., Jiang S., Bai W., Dai L., Jiang S., Hu J., Ren E., Guo R. (2023). Construction of hierarchical carbon fiber Aerogel@Hollow Co_9_S_8_ polyhedron for high-performance electromagnetic wave absorption at low-frequency. Chem. Eng. J..

[55] Yuan M., Zhao B., Which C., Pei K., Money L., Zhang R., You W., Liu X., Zhang X., Che R. (2022). Remarkable magnetic exchange coupling via constructing bi-magnetic interface for broadband lower-frequency microwave absorption. Adv. Funct. Mater..

[56] Du Y., Liu W., Qiang R., Wang Y., Han X., Ma J., Xu P. (2021). Shell thickness-dependent microwave absorption of core-shell Fe_3_O_4_@C composites. Chem. Eng. J..

[57] Lv H., Liang X., Cheng Y., Zhang H., Tang D., Zhang B., Ji G., Du Y. (2015). Coin-like α-Fe_2_O_3_@CoFe_2_O_4_ Core–Shell Composites with Excellent Electromagnetic Absorption Performance. ACS Appl. Mater. Interfaces.

[58] Hadjipanayis G., Kim A. (1988). Domain wall pinning versus nucleation of reversed domains in R-Fe-B magnets (invited). J. Appl. Phys..

[59] Jiang J., Li D., Geng D., An J., He J., Liu W., Zhang Z. (2014). Microwave absorption properties of core double-shell FeCo/C/BaTiO_3_ nanocomposites. Nanoscale.

[60] Lu B., Dong X., Huang H., Zhang X., Zhu X., Lei J., Sun J. (2008). Microwave absorption properties of the core/shell-type iron and nickel nanoparticles. J. Magn. Magn. Mater..

[61] Lim J., Garg A., Ketterson J. (2021). Ferromagnetic resonance modes in the exchange-dominated limit in cylinders of finite length. Phys. Rev. Appl..

[62] Lv H., Ji G., Liu W., Zhang H., Du W. (2015). Achieving hierarchical hollow carbon@Fe@Fe_3_O_4_ nanospheres with superior microwave absorption properties and lightweight features. J. Mater. Chem. C.

[63] Wang W., Zhang H., Zhao Y., Wang J., Zhao H., Li P., Yun J. (2021). A novel MOF-drived self-decomposition strategy for CoO@N/C-Co/Ni-NiCo_2_O_4_ multi-heterostructure composite as high-performance electromagnetic wave absorbing materials. Chem. Eng. J..

[64] Wang Z., Tao J., Zhang X., Wei B., Yao Z., Jiang H., Liu J., Zhou J., Tao X. (2023). Constructing FeCo@C core-shell structure with strong polarization behavior towards excellent microwave absorption performance. Mater. Chem. Phys..

