# Supplementary material for: Exploring the Microstructural Effect of FeCo Alloy on Carbon Microsphere Deposition and Enhanced Electromagnetic Wave Absorption
Source: Nanomaterials (Basel). 2024 Jul 12;14(14):1194. doi: 10.3390/nano14141194 (PMC11279823; doi:10.3390/nano14141194)
Supplement: Supplementary file 1 [file nanomaterials-14-01194-s001.zip › nanomaterials-3087974-supplementary.pdf]

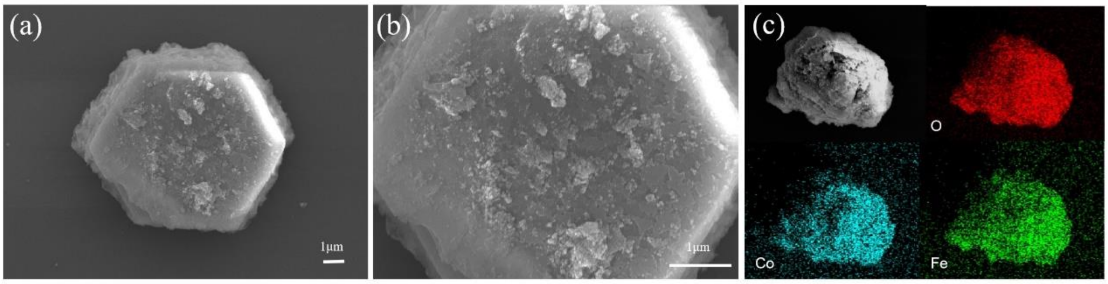


Figure S1. (a-b) SEM images and (c) EDS scanning element distribution map of $Fe(OH)_3/Co(OH)_2$ precursor.

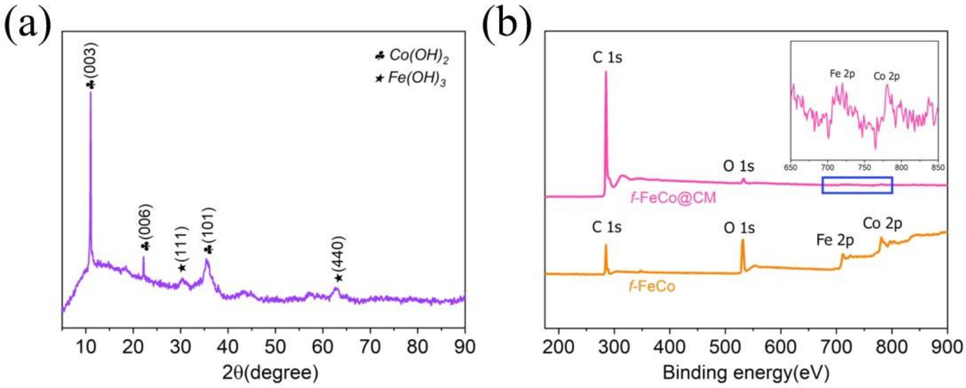


Figure S2. (a) XRD pattern of *f*-$Fe(OH)_3/Co(OH)_2$ precursor; (b) XPS spectra scan of *f*-FeCo and *f*-FeCo@CM samples.

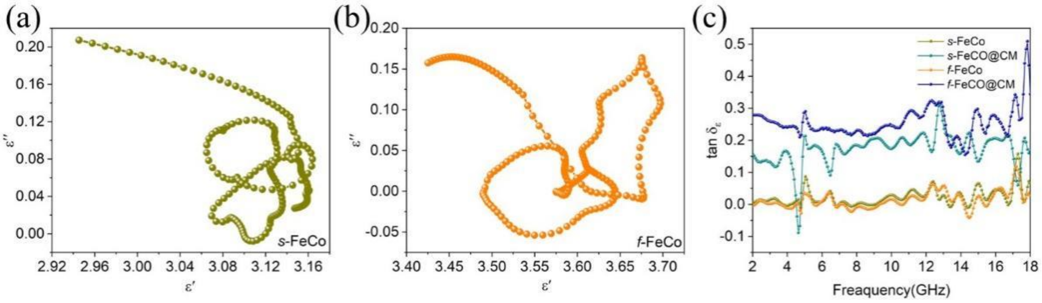


Figure S3. (a) $\varepsilon'$-$\varepsilon''$ curves of *s*-FeCo and (b) *f*-FeCo; (c) Dielectric loss tangent curves of different samples.

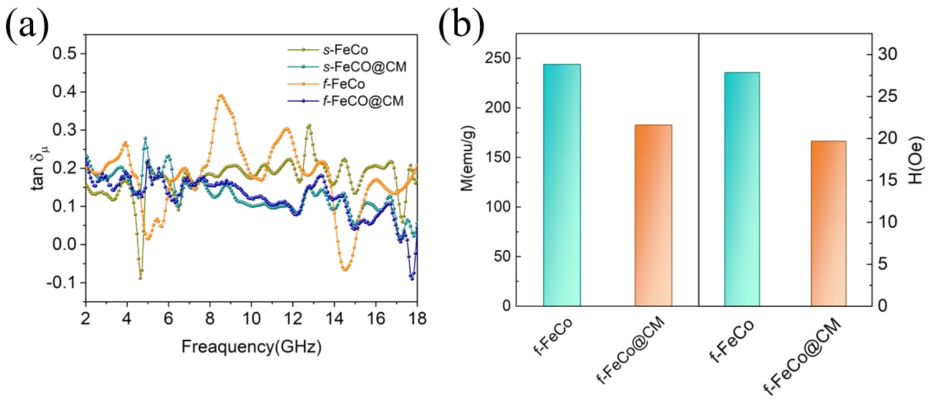


Figure S4. (a) Tangent curves of magnetic loss for different samples; (b) Bar chart of saturation magnetization ($M_s$) and coercivity ($H_c$) for *f*-FeCo and *f*-FeCo@CM samples.
